# Combined Multivariate Statistical Techniques and Water Quality Index (WQI) to Evaluate Spatial Variation in Water Quality

**DOI:** 10.21315/tlsr2023.34.3.7

**Published:** 2023-09-30

**Authors:** Shaheen Begum, Shahana Firdous, Zainab Naeem, Gul-e-Saba Chaudhry, Shanza Arshad, Fakiha Abid, Sania Zahra, Sehrish Khan, Muhammad Adnan, Yeong Yik Sung, Tengku Sifzizul Tengku Muhammad

**Affiliations:** 1 Department of Environmental Sciences, Fatima Jinnah Women University, Old Presidency, The Mall, Kachari Chowk, Rawalpindi 46000 Punjab, Pakistan; 2 Institute of Marine Biotechnology, Universiti Malaysia Terengganu, Kuala Nerus, 21030, Terengganu Malaysia; 3 Department of Botany, Kohat University of Science and Technology, Kohat-26000, Khyber Pakhtunkhwa, Pakistan

**Keywords:** Principal Component Analysis, PCA, Water Quality Index, WQI, Spatio Temporal, Base Cations, Eutrophication

## Abstract

In present study, Water Quality Index (WQI) has been assessed of the Rawal Lake which is a major source of drinking water for people in the Federal Capital, Islamabad, and its adjacent city Rawalpindi in Pakistan. For this, the principal component analysis (PCA) and WQI were applied as an integrated approach to quantitatively explore difference based on spatial variation in 11 water quality parameters of the five major feeding tributaries of the Rawal Lake, Pakistan. The results of temperature in water, total dissolved solids, pH, electrical conductivity, chlorides and sulfates were well within the allowable World Health Organisation’s (WHO) limits. However, the heavy metals like cadmium and lead were above permissible limits by the WHO in tributaries of Bari Imam and Rumli. Moreover, this has been proven by the Pearson correlation which suggested strong positive correlation (0.910*) between lead and cadmium. The results of present study were subjected to statistical analysis, i.e., PCA which gave three major factors contributing 96.5% of the total variance. For factor 1, pH, TDS, alkalinity, chlorides, sulfates and zinc have highest factor loading values (>0.60) and presented that these parameters were among the most significant parameters of first factor. As per the WQI results, the water was categorised in two major classes indicating that water of Bari Imam and Rumli is highly contaminated with heavy metals and totally unsuitable for drinking purposes. Based on the results of the present study, it is suggested to make heavy metals consideration as an integrated component in future planning for maintaining water quality of the Rawal Lake and its tributaries.

**Highlights**

- This study uses principal component analysis (PCA) and Water Quality Index (WQI) to assess Rawal Lake’s Water Quality Index (WQI) as a major source of drinking water for Islamabad and Rawalpindi in Pakistan.
- The study finds that while some parameters meet WHO limits, heavy metals like cadmium and lead exceed permissible limits in Bari Imam and Rumli tributaries, showing a strong positive correlation between lead and cadmium.
- Heavy metal considerations should be integrated into future planning to maintain water quality in Rawal Lake and its tributaries, as the water in Bari Imam and Rumli tributaries is highly contaminated and unsuitable for drinking purposes.

## INTRODUCTION

The poor water quality is a major issue for humans as it is directly connected with the mankind wellbeing (Iscen *et al*. 2008). Drinking water quality acts as a determinant for economic and social stability of a nation (Wu *et al*. 2018). Rivers and lakes are a source of drinking water and specifically in developing countries, these sources are being contaminated due to intense environmental pressure (Gad *et al*. 2022). Several natural and anthropogenic processes affect the drinking water quality and its utilisation (Todd *et al*. 2012). Water quality is influenced by both human activities and natural processes such as rock weathering, erosion and climate change. (Lashari *et al*. 2022). Furthermore, quality of drinking water also plays a critical role in maintaining optimum mineral concentration in humans which is important for the overall homeostasis (Mukate *et al*. 2019). Lakes and rivers are the most significant among freshwater reservoirs because of their essential and multiple utilisation including as sources of energy, drinking water, production, aquaculture and water for irrigation. All uses of lakes and streams are significantly contingent on quality of water. Therefore, water quality should be considered while planning new project which any way may interfere with the water quality. The hydrochemistry of natural waters is significantly influenced by the area’s climate and topography. Interaction between different factors determines chemical composition of water such as rock weathering in watershed, composition of soil and rain water, and chemical reactions between soil and water (Bengraïne *&* Marhaba 2003). The monitoring of water quality in lake is necessary to define the water geochemical nature that changes seasonally and is significantly influenced by anthropogenic activities (Şener *et al*. 2017). Healthy aquatic ecosystem is dependent on water quality determined by chemical, physical and biological properties of water (Thirupathaiah *et al*. 2012). WQI is utilised by policy makers globally to develop sound policies for water consumption and utilisation (Ponsadailakshmi *et al*. 2018). WQI enables the consolidation of large water quality datasets into a single, dimensionless index that is specifically designed with on-site specific guidelines (Uddin *et al*. 2021). WQI incorporates mathematical tools to assess different biological, chemical and physical properties that determine the overall water quality status (Abbasnia *et al*. 2019). Generally, drinking WQI is assessed in two steps; first step is selection of water quality parameters, and the other step involves selection of sub-indices which are then aggregated to evaluate the results (Adimalla *&* Venkatayogi 2018).

The spatial and seasonal water quality variations in the context of chemical properties are influenced at regional scales because of many factors (Begum *et al*. 2010; Rothwell *et al*. 2010; Wang *et al*. 2015). The water quality is significantly affected by inorganic pollutants like acids, salts, toxic metals; biological pollutants like protozoans, viruses and bacteria; various anions and cations like sulphates, phosphates, nitrates, calcium, magnesium and fluoride; and water-soluble substances emitting radiations (radioactive material). Both natural and anthropogenic sources contribute to the addition of heavy metals to water (Firdous *et al*. 2016; Midrar-Ul-Haq *et al*. 2005; Wang *et al*. 2015; Ilyas *&* Sarwar 2003). Moreover, water pollutants are derived from point and non-point sources however, major portion of pollutants are contributed by non-point sources (Yaseen *et al*. 2018). Aquatic heavy metal pollution has become one of the major targets of the world’s environmentalists due to toxic effects on human health and ecosystem stability (Koffi *et al*. 2014).

There is a critical water shortage and pollution in Pakistan like other under-developed countries. As per the International Monetary Fund (IMF), Pakistan is ranked on third number among the highly water stressed countries. Moreover, according to Pakistan Council of Research in Water Resources’ report of 2018, the clean water will be runout in the country by the year 2025 (Shukla 2018). The per capita availability of water has declined from 5,000 m^3^ per annum in 1950s to below 1,000 m^3^ in 2018 and will drop further to 860 m^3^ by the year 2025 (Aziz *et al*. 2018). Surface water quality deterioration in Pakistan is consistent like other developing countries (Malik *&* Zeb 2009). Also, 80% of the population of Pakistan has no or limited access to clean drinking water (Daud *et al*. 2017). Urbanisation, increasing population, the expansion of industries, run off from agricultural areas and discharges from domestic sources into natural water reservoirs from the watersheds pollute surface and subsurface freshwater water (Ahad *et al*. 2006; Tariq *et al*. 2008; Muhammad *et al*. 2010; Azizullah *et al*. 2011). Although the National Water Policy (Ministry of Water Resources, Government of Pakistan 2018) recognises the looming crisis of drinking water quality, yet due to more demand and less monitoring of water quality, the crises are being exacerbated (Nabi *et al*. 2019).

Rawal Lake, which is an artificial reservoir of Pakistan, created in 1962 by Water and Power Development Authority (WAPDA) covers an area of 8.82 km^2^ and is a drinking water source for twin cities of Islamabad and Rawalpindi (Malik *et al*. 2017). This lake is currently plagued by numerous issues, including silting, effluent discharge, environmental degradation, water pollution and overfishing (Chandio *et al*. 2019). A severe heavy metal pollution of surface water has been reported in water samples and in fish samples (*C. carpio*) collected from Rawal Lake (Malik *&* Nadeem 2011; Iqbal *&* Shah 2014). High concentration of heavy metals including lead, copper and chromium has been reported in water samples from Rawal Lake (Malik *et al*. 2014). Contamination in the Rawal Lake is caused by the encroachment and unplanned human-settlement development in the studied area (Rawal Lake Catchment). The Rawal Lake is critically polluted by the release of sediments and untreated waste from domestic sources including Quaid-e-Azam University, Nurpur Shahan, Bani Gala, Bhara Kahu, Malpur, Diplomatic Enclave and Ghora Gali (Aftab 2010; Firdous *et al*. 2016). The present study was planned for the assessment of water quality through physico-chemical parameters determination in the major contributing tributaries of the Rawal Lake, Islamabad. Statistical analysis of data helped in isolating the most influencing variables of water quality.

## METHODOLOGY

### Study Sites and Sampling Methods

**Figure 1:**
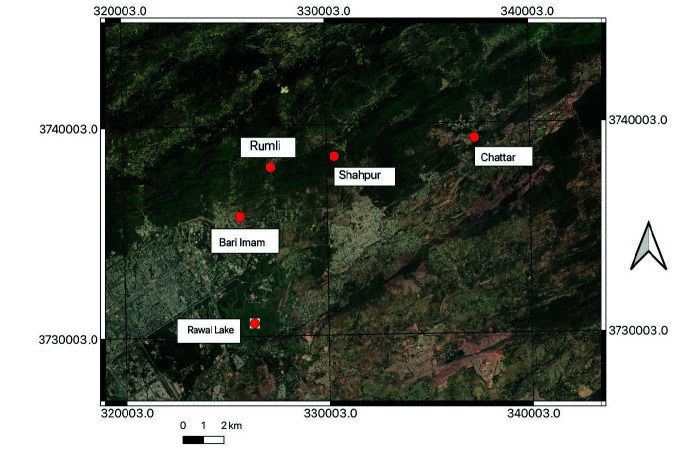
Map of the study area showing locations of sampling points.

The present research work was conducted in different tributaries (Bari Imam, Chattar, Rumli near Quaid-i-Azam University, Shahdra and Shahpur) of the Rawal Lake in capital city of Islamabad, Pakistan (Fig. 1). The water samples were collected in pre-rinsed clean polyethylene bottles directly from these sites of the Rawal Lake using water samplers.

To ensure representative samples, water was filled up to 90% in the bottles and were shaken before analysis to ensure mixing. For quality control and quality assurance, standard procedures were adopted such as bottles were pre-washed using deionised water and these bottles were properly labeled with sampling code, source, date and time of collection (Gav *et al*. 2018). The bottles were stored in a cool place and were transferred to the laboratory of Environmental Sciences department for further analysis. The samples were kept in the refrigerator at 4°C prior to analysis (Table 1).

The sampling was performed in March and then repeated in May and July. The total of 12 samples were randomly collected from each tributary in each sampling month. Shahdra and Chattar were recreational points while the pollution in Bari Imam is attributed to high population-density, household waste dumping and car wash activities. In the field visits grassland, forestland and agricultural areas were observed in the Rumli site, however Shahpur majorly consisted of barren land.

### Parameters Studied and Laboratory Analysis

Water temperature was measured by thermometer. pH was determined by WTW series 720 pH meter and Jenway 470 portable conductivity meter was used to measure TDS and EC of water samples. Alkalinity was measured by titrating the samples against acid. Heavy metals were determined by digesting each water samples with 10 mL nitric acid and then analysis was done by using Perkin-Elmer Analyst 300 Atomic Absorption Spectrophotometer (Olaifa *et al*. 2004; Lokeshwari & Chandrappa 2006).

### Statistical Analysis

The water sampling was conducted thrice in three months under the principle of Randomised Complete Block Design (RCBD) in which field replicates were collected for mean statistical analyses. PCA was executed on 10 variables of 5 sampling sites in 3 sampling months for the identification of significant parameter of water quality using XLSTAT. For understanding the variation pattern in quality of water, the lake water quality dataset was subjected to multivariate techniques. Pearson correlation with statistical significance at *p* < 0.05 was used to study relationships among water quality parameters using SPSS version 13. The PCA was employed on a dataset to define data set interpretation, data reduction and to find out statistical correlation between water parameters with minimum loss of original information (Kazi *et al*. 2009). The aforementioned approach is useful for the interpretation and modelling of large datasets to reduce dimensionality in data for yielding useful information in the context of water quality assessment and management (Simeonov *et al*. 2003).

### Water Quality Index (WQI)

WQI is among the most effective tool for the provision of comprehensive status of the water quality. It is defined as rating of individual water-quality parameters that reveals composite influence of parameters and it is calculated to assess water suitability for human use. Three steps were followed for computing WQI (Ravikumar *et al*. 2013). In first step, a relative weight (*w_i_*) was assigned to each of the parameter according to its observed effects on primary health and its importance in overall drinking water quality. The range of weight was from 1–5 and maximum weight is assigned to those parameters which have critical health effects and whose concentration above the permissible limit could limit usability of the water for domestic and drinking purposes (Yidana *et al*. 2010). The second step (Equation 1) was the calculation of relative weight of each studied parameter (Table 2).


(1) $$W_{i}=\frac{W_{i}}{\Sigma_{i=1}^{n}},$$

Where *W_i_* is the relative weight, *w_i_* is the weight of each parameter while *n* is the total number of parameters.

In third step, for each parameter the quality rating (*q_i_*) was calculated by dividing concentration of each water quality parameters by its respective standard proposed by WHO (WHO 2004).


(2) $$q_{i}=(C_{i}S_{i})\times 100$$

Where *q_i_* is quality rating_;_ *C_i_* is concentration of each parameter and *S_i_* is the drinking water standard set by WHO for each parameter. Units of all parameters determined in present study and WHO standards were kept the same for consistency. To compute WQI, SI is first determined for each parameter.


(3) $$\text{S}1=w_{i}q_{i}$$

(4) $$\text{WQI}=\sum {\text{SI}}_{\text{i}}$$

Where SI is the sub index computed based on relative weight and concentration rating of each parameter and WQI is summation of SI sub index of *i-*th parameter.

## RESULTS AND DISCUSSION

The lake and its tributaries are a source of drinking water for the residents of Islamabad and its adjoining towns and the twin city of Rawalpindi. A large volume of population depends upon this source of water hence, it is imperative upon the local government to ensure that the water quality of the lake is suitable for drinking by the residents. As polluted water becomes a source of illness, the health of the residents of federal capital purely depends upon this lake. The following data can be utilised by the government and the policy makers to develop mechanism of avoiding pollution in the lake. Moreover, the data can also be used by health care authorities to assess if the public is getting affected due to the polluted water. The results of the present research are discussed in detail in the following headers.

### Temperature

**Figure 2:**
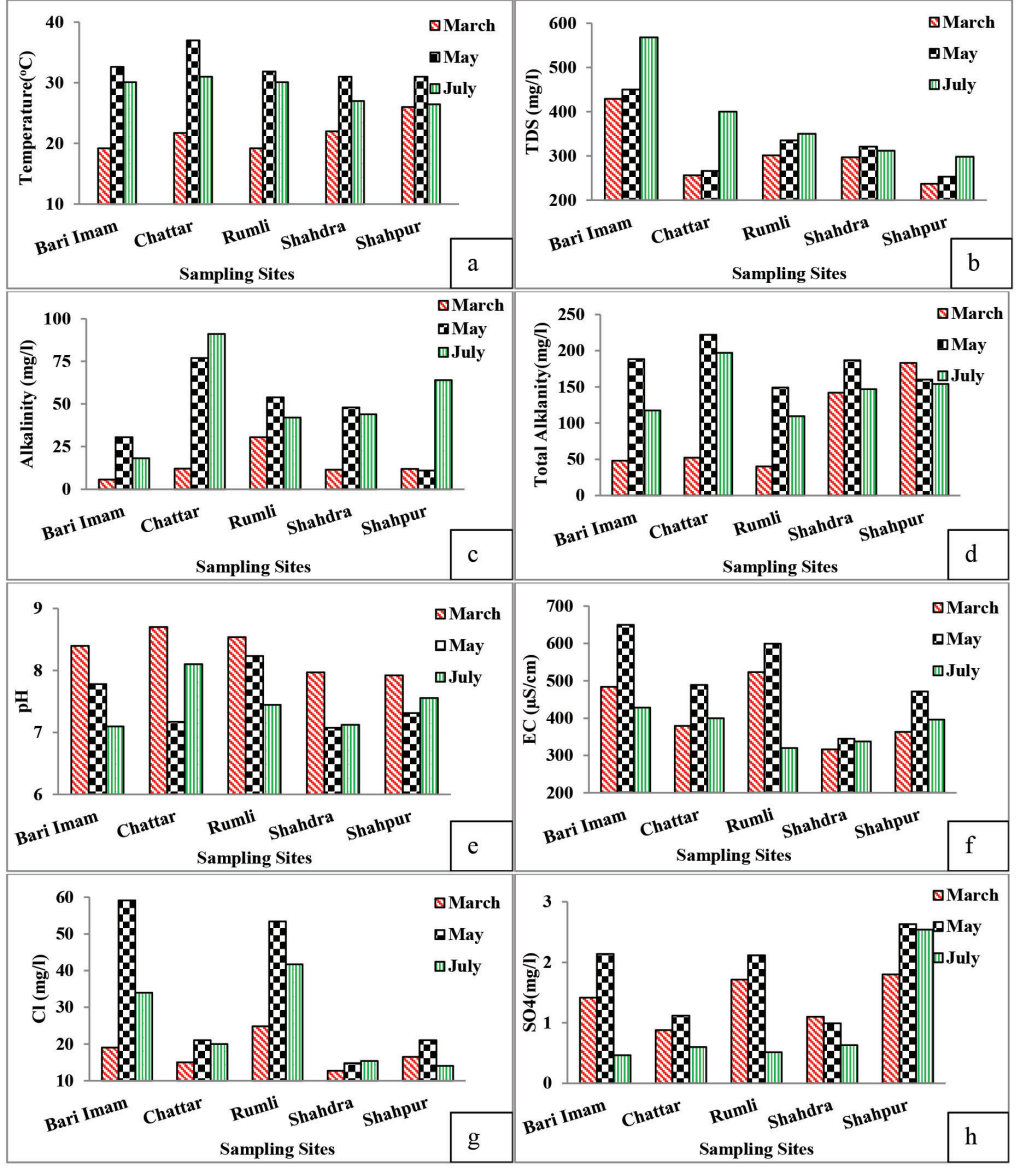
Results of different parameters measured in water samples collected from Bari Imam, Chattar, Rumli, Shahdra and Shahpur in March, May and July.

Temperature ranged from 19°C to 37°C from March to July (Fig. 2a). Chattar stream had highest temperature among all other sites. Maximum temperature was observed in May. According to Poole and Berman (2001), water temperatures are dynamic over space and time as the present study showed that the temperature was lowest in March as the water samples in March were mostly collected in the evening, so the temperature was lower. Moreover, summer temperatures and winter precipitation greatly affect the overall temperature of lake water (Collins *et al*. 2019). Variations in water temperature often follow daily fluxes in solar heating (Stoneman *&* Jones 1996). According to Salve and Hiware (2008), higher temperature in warmer months is attributed to low water level, high air temperature and clear atmosphere. The increase in total dissolved solids in the month of May and July may be also responsible for the increase in temperature as according to Phiri *et al*. (2005), total dissolved solids are responsible for absorbing heat. Temperature was within permissible limits of WHO, NEQS and PSI (Table 3).

### Total Dissolved Solids (TDS)

Spatial and temporal variation in TDS is related to natural factors like seasonal effects and flow regime, and man-made impacts too (Voutsa *et al*. 2001). Also, during rainy season, TDS concentration is low due to dilution effect in comparison to dry season (Umer *et al*. 2020). In present study TDS ranged from 237 mg/L to 568 mg/L from March to July in all the sampling sites (Fig. 2b). Highest value of TDS was observed in May and in Bari Imam. Water samples had more TDS in May and July due to rainfall and water runoff from the respective catchments of the water tributaries. The bicarbonate, carbonate, sulphate, chloride, nitrates and other substances contribute mainly to the formation of TDS (Shaikh & Mandre 2009). According to Sarwar *et al*. (2008), the main sources of TDS in water are rock, soils, precipitation, wind and household wastes of human and livestock. Total dissolved solids were within permissible limits proposed by WHO, NEQS and PSI.

### Alkalinity

Alkalinity of water is defined as the presence of all such substances in water which have the ability to resist the pH change upon the addition of an acid to water. Lake water alkalinity is greatly affected by the microenvironment and the availability of sunlight in the lake layers (Søndergaard *et al*. 2020). Spatio temporal variation in water alkalinity was observed in different tributaries of the Rawal Lake like other parameters. Alkalinity ranged from 5.75 mg/L to 91 mg/L (Fig. 2c). Chattar had highest values of alkalinity while Bari Imam had lowest values. Lower values of alkalinity were observed in March while higher values were observed in July.

### Total Alkalinity

Total alkalinity of water is due to carbonate and bicarbonate anions. Total alkalinity ranged from 40 mg/L to 222 mg/L (Fig. 2d). Total alkalinity was lower in March whereas in May and June its values were high. Chattar had higher values of alkalinity than other sites. The total alkalinity values were maximum in summer due to increase in bicarbonates in the water.

### Water pH

Water pH of the study area was found to be alkaline in nature ranging from 7.075 to 8.7 (Fig. 2e). According to WASA (2011) report, geological basis of Rawalpindi and Islamabad are composed of sedimentary rocks containing more limestone, so the liming effect of limestone raises the pH of water. As a result, pH of the study area was slightly alkaline. Maximum value of pH was observed in March. As march was drier month with no rainfall and had more evaporation of water leaving behind salts in the water that caused an increase in the pH while there was a heavy rainfall in May and July that lead to the decrease in pH as according to study of Shinde *et al*. (2011), lower pH values of water are attributed to dilution caused by the rainwater.

Rumli and Chattar streams have higher values of pH among all other sampling sites. Higher pH values can be attributed to steepness of the area or the run off from agricultural sites of Rumli to the water streams and addition the base cation in the stream water. The pH findings of present study are supported by the research of (Templer *et al*. 2005) who investigated that agricultural soils have comparatively less soil pH and lower base cations concentrations due to larger losses during runoff that in turn increases the pH of the water. Values of pH were within permissible limits of WHO, NEQS and PSI (Table 3).

### Electrical Conductivity

Electrical conductivity of the sampling sites ranged from 316 μS/cm to 650 μS/cm from March to July (Fig. 2f). Overall, Bari Imam and Rumli had higher values of EC than other tributaries of the Rawal Lake. Highest value of EC was observed in May. Pearson correlation resulted in strong positive correlation between temperature and electrical conductivity (*r* = 0.819) indicating that warmer the water, higher the conductivity.

Temporal variation in electrical conductivity was attributed to changes in temperature. 1°C increase in temperature can increase electrical conductivity by 1.9% (Corwin *&* Lesch 2003; McCutcheon *et al*. 2006). High conductivity values in the water of Bari Imam are attributed to the nearby dumpsite that is an indication of adverse effects on water quality (Basha *et al*. 2014). Values of EC were slightly higher than the permissible limits of WHO (Table 3).

### Chlorides

Concentration of chlorides in water of the sampling tributaries of the Rawal Lake ranged from 12.7 mg/L to 59.1 mg/L (Fig. 2g). Increasing order of chloride was seen in different studied months, i.e., March < July < May. Higher values of chlorides were recorded in Bari Imam and Rumli as both these sites were more polluted than other sampling sites. Bari Imam and Rumli were also used for animal grazing and according to Mahananda *et al*. (2010), higher level of chlorides in the water may be due to organic pollution of animal origin, human feces, sewage inflow and weathering of sedimentary rocks. According to Yahya *et al. (*2012), higher level of chloride in dry season is due to cations evaporation of water, and higher level of chloride after rainfall is associated with eutrophication in streams and lakes and in present study drier month March had lower values of chlorides than other sampling months. Chlorides were within permissible limits of WHO, PSI and NEQS.

### Sulfates

Sulfates ranged from 0.4 mg/L to 2.63 mg/L from March to July (Fig. 2h). Industrial waste is the major discharge source of sulphate in the aquatic environment. The most common industrial source of sulphase include wastes from mining and smelting operations, kraft pulp and paper mills, textile mills and tanneries. Sulphate in the natural water is about 5 mg/L–50 mg/L. The concentration of sulphate for smooth functioning of the aquatic organisms should not exceed above 250 mg/L (Khan et al. 2014). Values of sulfates in the present study were much less than permissible limits set by WHO, NEQS and PSI (Table 3).

### Heavy Metals

The main attributing natural source of heavy metals in water is mineral weathering. While in aquatic ecosystem the total heavy metal content demonstrate the status of current pollution of the studied area (Arnous & Hassan 2015). It was reported in the current study that heavy metal concentrations in Shahdra and Chattar streams were less than the detection limits while zinc in the studied water samples of Shahpur stream ranged from 0.017 mg/L to 0.331 mg/L. The concentration of Cd and Pb in water samples of Rumli stream ranged from 0.025 mg/L to 0.431 mg/L and 0.081 mg/L to 0.95 mg/L, respectively. In Bari Imam stream, Cd ranged from 0.008 mg/L to 0.031mg/L. The amount of zinc reported was within the allowable limits in all studied sites while Cd and Pb were above the permissible limits of WHO, NEQS and PSI, respectively (Table 3). Waste release from domestic and industrial sources such as from smelting processes, automobiles and mining activities contribute Pb to the environment. The combustion of gasoline is the major and significant man-made source of Pb in the environment and specifically water (Gill *et al*. 2013). Paints, sewage, fertilizers and pesticides are other human originated sources adding pollution load in water system (Qadi *et al*. 2008).

To determine the common source of metals in water, a correlation coefficient was also calculated for heavy metals. Cd and Pb showed statistically strong (positive) correlation (*r* = 0.910*) (Table 4). Strong correlation between Cd and Pb indicated that both these metals share a common source and were recognized as outputs from municipal sewage system. On the other hand, strong correlation can also be justified by the fact that Cd is present as natural impurity in Zn and Pb ores (Morrow 2001; Bhuiyan *et al*. 2015).

### Principal Component Analysis (PCA)

The objective of PCA was to generate new sets of factors or components much smaller in number than the original data set of parameters (Iscen *et al*. 2008). The factor loadings, eigenvalues, percentage variance and cumulative percentage variance are given in Table 5. Liu *et al*. (2003) classified factor loadings as weak, moderate and strong corresponding to absolute loading values of 0.30–0.50, 0.50–0.75 and > 0.75, respectively. PCA was executed on 10 variables of 5 sampling sites in 3 sampling months for the identification of significant parameter of water quality. Eigenvalue represents a measure of level of significance of a component or factor. Eigenvalues of 1 or greater are considered significant (Loska *&* Wiechuła 2003).

Three major factors were achieved by the application of PCA in the present study, contributing 96.59% of the total variance. Factor 1 (F1), namely physicochemical factors, explained 43.6% of the total variance and has strong positive loadings of TDS, chlorides, sulfates and zinc and strong negative loadings of pH and alkalinity. The second factor related to second eigenvalue (3.879) reported for 32% of the variance with positive loading of temperature, EC, sulfates, cadmium, lead and negative loading of TDS. F2 indicated strong associations of Cd and Pb. The studied toxic heavy metals are attributed to enter in the water ecosystem through runoff coming from agricultural wastewater, the deposition of atmospheric pollutants and emissions from vehicles respectively (Iqbal, Shah & Akhter 2013; Iqbal, Tirmizi and Shah 2013). Third factor with eigenvalue (2.476) accounted 20.6% of the total variance.

### Water Quality Index (WQI)

Water is classified into the following five categories on the basis of WQI values (Sahu *&* Sikdar 2008): < 50 = excellent water; 50–100 = good water; 100–200 = poor water; 200–300 = very poor water; >300 = water unsuitable for drinking.

Results indicated that 75% and 87% water samples of Chattar, and Shahdra fall under excellent water quality, respectively. While 50% samples of Shahpur had good quality water. However, in Bari Imam and Rumli, more than 50% samples were of poor quality even unsuitable for drinking purpose (Table 6).

## CONCLUSION

The conclusion of present research showed that except the heavy metals, most of the water quality parameters of the studied five selected tributaries of the Rawal Lake were within allowable ranges of various water-quality standards. The lead and cadmium contents were above the allowable limits, which are attributed to the direct release of waste in stream water specifically at Bari Imam tributary. Those parameters which were responsible for water quality variation were identified through the application of PCA. WQI indicated that water of Bari Imam and Rumli were unfit for various uses specifically drinking as well as irrigation owing to the higher quantity of the studied heavy metals. Heavy metals presence in higher quantity may have negative impact on human, plants and animals’ health. Based on the results of present study, it is suggested to execute water quality studies over longer period of time to check out the effects of pollutants on water quality. Analysis of soil chemistry in relation water chemistry is further suggested to enhance the current understanding of the study area to provide base line data for the development of integrated management plan.

## Figures and Tables

**Table 1:** Preservation methodologies for different parameters (Maiti 2011).

Parameters	Preservative	Maximum holding time
Alkalinity	Cool, 4°C	24 hours
Chloride	Cool, 4°C	7 days
pH	None	6 hours
Nitrate	Cool, 4°C	24 hours
Sulphates	Cool, 4°C	7 days
EC	Cool, 4°C	
Heavy metals		
Lead	1 mL conc. Nitric acid/L sample	6 months
Chromium	1 mL conc. Nitric acid/L sample	6 months
Cadmium	1 mL conc. Nitric acid/l sample	6 months
Zinc	1 mL conc. Nitric acid/L sample	6 months
Copper	1 mL conc. Nitric acid/L sample	6 months

*Note*: conc. = concentrated

**Table 2:** Relative weight of each studied chemical parameter.

Parameters	WHO limits	Weight (*w_i_*)	Relative weight (*W_i_*)
pH	7.7	3	0.1
EC	500	3	0.1
Alkalinity	275	2	0.067
TDS	1,000	5	0.167
SO_4_	300	3	0.1
Cl	250	3	0.1
Pb	0.01	5	0.167
Zn	3	1	0.033
Cd	0.003	5	0.167

		**∑*w****_i_* = 30	**∑*W****_i_* = 1

*Notes*: *W_i_ =* relative weight; w_i_ = weight of each parameter.

**Table 3:** Range and average values of parameters and their comparison with drinking water standards of WHO, NEQS and PSI.

Parameters	Sites	Range	Average	WHO	NEQS	PSI
Temperature (°C)	Bari Imam	19–32	27	40	40	40
	Chattar	22–37	29			
	Rumli	19–32	26			
	Shahdra	22–31	27			
	Shahpur	26–31	28			
pH	Bari Imam	7.1–8.3	7.7	6.5–9.0	6–10	6.5–9.2
	Chattar	7.1–8.7	7.9			
	Rumli	7.4–8.5	8.0			
	Shahdra	7.1–7.9	7.4			
	Shahpur	7.3–7.9	7.6			
EC (μS/cm)	Bari Imam	428–650	528	500		
	Chattar	379–489	427			
	Rumli	319–600	472			
	Shahdra	316–344	332			
	Shahpur	363–471	417			
Alkalinity (mg/L)	Bari Imam	5.7–30	18	50–500		
	Chattar	12–91	56			
	Rumli	30–53	42			
	Shahdra	11–48	32			
	Shahpur	11–64	33			
TDS (mg/L)	Bari Imam	429–568	488	1,000	500– 3,500	1500
	Chattar	256–400	315			
	Rumli	301–350	327			
	Shahdra	297–321	309			
	Shahpur	237–298	264			
SO_4_ (mg/L)	Bari Imam	0.4–2.1	1.3	200–400	600	200–400
	Chattar	0.6–1.1	0.9			
	Rumli	0.5–2.1	1.4			
	Shahdra	0.6–1.1	0.9			
	Shahpur	1.8–2.7	2.3			
Cl (mg/L)	Bari Imam	19–59	38	250	1,000	200–600
	Chattar	15–21	18			
	Rumli	24–53	39			
	Shahdra	13–15	14			
	Shahpur	14–21	17			
Pb (mg/L)	Bari Imam	0.02–1.2	0.62	0.01	0.5	0.05
	Chattar	BDL	BDL			
	Rumli	0.08–0.95	0.19			
	Shahdra	BDL	BDL			
	Shahpur	0.002–0.067	0.009			
Zn (mg/L)	Bari Imam	0.08–2.43	1.35	3	5.0	5
	Chattar	BDL	BDL			
	Rumli	0.9–1.3	1.15			
	Shahdra	BDL	BDL			
	Shahpur	0.02–0.33	0.10			
Cd (mg/L)	Bari Imam	0.008–0.031	0.018	0.003	0.01	0.01
	Chattar	BDL	BDL			
	Rumli	0.03–0.43	0.09			
	Shahdra	BDL	BDL			
	Shahpur	BDL	BDL			

**Table 4:** Pearson’s correlation results of studied water quality parameters based on average values of each study site.

	Temp (**°**C)	pH	EC (μS/cm)	TDS (mg/L)	Alkalinity (mg/L)	Total alkalinity (mg/L)	Chlorides (mg/L)	Sulfates (mg/L)	Zn (mg/L)	Cd (mg/L)	Pb (mg/L)
Temp (**°**C)	1	−0.923	0.819*	0.418	0.858	0.890*	0.973	0.183	−0.076	0.481	0.832
pH		1	−0.201	0.194	0.305	0.305	−0.010	−0.864*	0.686	−0.781	−0.724
EC (μS/cm)			1	0.917*	0.058	0.627	0.939*	0.460	0.747	−0.454	0.821
TDS (mg/L)				1	−0.297	0.280	0.898*	0.010	0.184	−0.409	0.065
Alkalinity (mg/L)					1	0.998*	0.726	0.409	0.267	0.863	−0.045
Total alkalinity (mg/L)						1	−0.562	0.224	−0.406	−0.215	0.525
Chlorides (mg/L)							1	0.534	0.987*	0.226	0.991
Sulfates (mg/L)								1	0.545	−0.673	0.642
Zn (mg/L)									1	0.295	0.473
Cd (mg/L)										1	0.910*
Pb (mg/L)											1

*Notes*: Values in bold are different from 0 with a significance level alpha = 0.05

**Table 5:** Factor loading of each parameter in three factors with their percent variance and eigenvalues.

Parameters	F1	F2	F3
Temperature (**°**C)	0.487	0.745	0.354
pH	−0.922	−0.054	0.368
EC (μS/cm)	0.200	0.774	−0.547
TDS (mg/L)	0.609	−0.539	−0.580
Alkalinity (mg/L)	−0.890	0.336	−0.044
Total alkalinity (mg/L)	−0.882	0.318	−0.344
Chlorides (mg/L)	0.952	0.191	−0.133
Sulfates (mg/L)	0.845	0.512	−0.081
Zinc (mg/L)	0.703	−0.253	0.621
Cadmium (mg/L)	0.110	0.945	0.254
Lead (mg/L)	−0.201	0.976	0.073
Eigenvalue	5.237	3.879	2.476
Variability (%)	43.639	32.323	20.631
Cumulative (%)	43.639	75.962	96.592

**Table 6:** Calculation of WQI and indication of water type of individual water samples of sampling sites of the Rawal Lake.

Sample no.	Bari Imam		Chattar		Rumli		Shahdra		Shahpur	
				
	WQI	Water type	WQI	Water type	WQI	Water type	WQI	Water type	WQI	Water type
1	96	Good water	31	Excellent water	54	Good water	35	Excellent water	41	Excellent water
2	81	Good water	36	Excellent water	47	Excellent water	25	Excellent water	139	Poor water
3	196	Poor water	79	Good water	75	Good water	54	Good water	86	Good water
4	144	Poor water	68	Good water	238	Very poor water	34	Excellent water	79	Good water
5	2,052	Unsuitable for drinking	38	Excellent water	173	Poor water	45	Excellent water	65	Good water
6	736	Unsuitable for drinking	39	Excellent water	493	Unsuitable for drinking	67	Good water	36	Excellent water
7	369	Unsuitable for drinking	38	Excellent water	2,111	Unsuitable for drinking	48	Excellent water	97	Good water
8	1,232	Unsuitable for drinking	40	Excellent water	687	Unsuitable for drinking	31	Excellent water	37	Excellent water
